# Pathogens distribution and antimicrobial resistance in bloodstream infections in twenty-five neonatal intensive care units in China, 2017–2019

**DOI:** 10.1186/s13756-021-00989-6

**Published:** 2021-08-16

**Authors:** Jing Liu, Zengyu Fang, Yonghui Yu, Yanjie Ding, Zhijie Liu, Chengyuan Zhang, Haiying He, Hongli Geng, Weibing Chen, Guoying Zhao, Qiang Liu, Baoying Wang, Xueming Sun, Shaofeng Wang, Rongrong Sun, Delong Fu, Xinjian Liu, Lei Huang, Jing Li, Xuexue Xing, Xiaokang Wang, Yanling Gao, Renxia Zhu, Meiying Han, Fudong Peng, Min Geng, Liping Deng

**Affiliations:** 1 Department of Neonatology, Shandong Provincial Hospital Affiliated to Shandong First Medical University, Jinan, 250021 Shandong China; 2 Department of Neonatology, Shandong Provincial Hospital, Cheeloo College of Medicine, Shandong University, No. 234, Jingwu Road, Huai Yin District, Jinan, 250021 Shandong China; 3 Department of Pediatrics, Yantai Yuhuangding Hospital, Yantai, 264000 Shandong China; 4 Department of Neonatology, The First Affiliated Hospital of Shandong First Medical University, Jinan, China; 5 Department of Neonatology, Weifang Maternal and Child Health Hospital, Weifang, China; 6 Department of Pediatrics, Baogang Third Hospital of Hongci Group, Baotou, China; 7 Department of Neonatology, Zibo Maternal and Child Health Hospital, Zibo, China; 8 Department of Pediatrics, People’s Hospital of Rizhao, Rizhao, China; 9 Department of Pediatrics, Binzhou Medical University Hospital, Binzhou, China; 10 Department of Pediatrics, Linyi People’s Hospital, Linyi, China; 11 Department of Pediatrics, Women and Children’s Health Care Hospital of Linyi, Linyi, China; 12 Department of Pediatrics, Weifang Yidu Central Hospital, Weifang, China; 13 Department of Neonatology, Jinan Maternity and Child Health Care Hospital, Jinan, China; 14 Department of Pediatrics, Dongying People’s Hospital, Dongying, China; 15 Department of Pediatrics, Tengzhou Central People’s Hospital, Tengzhou, China; 16 Department of Pediatrics, Hebei Petro China Central Hospital, Langfang, China; 17 Department of Neonatology, Shandong Provincial Maternity and Child Health Care Hospital, Jinan, China; 18 Department of Pediatrics, The Second Affiliated Hospital of Shandong First Medical University, Taian, China; 19 Department of Pediatrics, Jinan Central Hospital, Cheeloo College of Medicine, Shandong University, Jinan, China; 20 Department of Pediatrics, Shandong Provincial Hospital Affiliated to Shandong First Medical University, Jinan, China; 21 Department of Pediatrics, Dezhou People’s Hospital, Dezhou, China; 22 Department of Pediatrics, Zibo Central Hospital, Zibo, China; 23 Department of Pediatrics, Liaocheng People’s Hospital, Liaocheng, China; 24 Department of Pediatrics, The Second People’s Hospital of Liaocheng, Liaocheng, China; 25 Department of Neonatology, The Second Children and Women’s Healthcare of Jinan City, Jinan, China; 26 Department of Pediatrics, Heze Municipal Hospital, Heze, China

**Keywords:** Neonatal sepsis, Antimicrobial resistance, Neonatal intensive care unit, *Klebsiella pneumoniae*

## Abstract

**Background:**

Overcrowding, abuse of antibiotics and increasing antimicrobial resistance negatively affect neonatal survival rates in developing countries. We aimed to define pathogens and their antimicrobial resistance (AMR) of early-onset sepsis (EOS), hospital-acquired late-onset sepsis (HALOS) and community-acquired late-onset sepsis (CALOS) in 25 neonatal intensive care units (NICUs) in China.

**Study design:**

This retrospective descriptive study included pathogens and their AMR from all neonates with bloodstream infections (BSIs) admitted to 25 tertiary hospitals in China from January 1, 2017, and December 31, 2019. We defined EOS as the occurrence of BSI at or before 72 h of life and late-onset sepsis (LOS) if BSI occurred after 72 h of life. LOS were classified as CALOS if occurrence of BSI was ≤ 48 h after admission, and HALOS, if occurrence was > 48 h after admission.

**Results:**

We identified 1092 pathogens of BSIs in 1088 infants from 25 NICUs. Thirty-two percent of all pathogens were responsible for EOS, 64.3% HALOS, and 3.7% CALOS. Gram-negative (GN) bacteria accounted for a majority of pathogens in EOS (56.7%) and HALOS (62.2%). The most frequent pathogens causing EOS were *Escherichia coli* (27.2%) and *group B streptococcus* (*GBS*; 14.6%) whereas in CALOS they were *GBS* (46.3%) and *Staphylococcus aureus* (41.5%). *Klebsiella pneumoniae* (27.9%), *Escherichia coli* (15.7%) and *Fungi* (12.8%) were the top three isolates in HALOS. Third-generation cephalosporin resistance rates in GN bacteria ranged from 9.7 to 55.6% in EOS and 26% to 63.3% in HALOS. Carbapenem resistance rates in GN bacteria ranged from 2.7 to 31.3% in HALOS and only six isolates in EOS were carbapenem resistant. High rates of multidrug resistance were observed in *Klebsiella pneumoniae* (60.7%) in HALOS and in *Escherichia coli* (44.4%) in EOS. All gram-positive bacteria were susceptible to vancomycin except for three *Enterococcus faecalis* in HALOS. All-cause mortality was higher among neonates with EOS than HALOS (7.4% VS 4.4%, [OR] 0.577, 95% CI 0.337–0.989; *P* = 0.045).

**Conclusions:**

*Escherichia coli*, *Klebsiella pneumoniae* and *GBS* were the leading pathogens in EOS, HALOS and CALOS, respectively. The high proportion of pathogens and high degree of antimicrobial resistance in HALOS underscore understanding of the pathogenesis and emphasise the need to devise effective interventions in developing countries.

**Supplementary Information:**

The online version contains supplementary material available at 10.1186/s13756-021-00989-6.

## Background

Neonatal bloodstream infection (BSI) is the third most common cause of neonatal morbidity and mortality globally, and is an ongoing major global public health challenge [1, 2]. Asia and Africa have the highest burden of BSIs in the world [2]. Scarcity in resources, insufficient surveillance and infection control, abuse of antibiotics and increase of antimicrobial resistance in low-income and middle-income countries (LMICs) may contribute to this situation [3, 4]. The risk of emergence and spread of antibiotic resistance in South East Asia is thought to be among the highest among all the WHO regions [5, 6]. Monitoring resistance in disease causing pathogens is of particular importance for neonatal BSIs in LMICs, where most treatments are empirically prescribed but should be based on reliable contemporaneous resistance data.

Neonatal sepsis was classified into early onset sepsis (EOS) and late onset sepsis (LOS) routinely [7, 8]. EOS generally reflects vertical transmission from mothers while LOS cases were likely due to pathogens acquired after delivery and often from nosocomial infections [9]. A previous meta-analysis reported that *Staphylococcus* species, especially *Coagulase negative Staphylococcus* (*CoNS*) continue to be the principal organisms of neonatal sepsis in China [10]. Nevertheless, more recent data described *Klebsiella pneumoniae* as the most frequent pathogen, with widespread antimicrobial resistance (AMR) [11]. *Klebsiella pneumoniae* was primarily associated with LOS, greater morbidity, mortality and limited treatment options in neonates [11, 12]. In China, multicenter reports on pathogens of neonatal BSI were scarce and were limited to specific gestational age groups or not involving resistance analysis of antibiotics [12, 13]. Currently, data on antimicrobial resistance (AMR) distinguishing between community-acquired LOS (CALOS) and hospital-acquired LOS (HALOS) in neonates are also scarce [3, 14]. Detecting emerging resistance in neonatal BSI is vital in order to optimise empiric antibiotic therapy in HALOS, in accordance with antimicrobial stewardship principle and to reduce mortality. The present study assessed the benchmark in neonatal sepsis, distinguishing between EOS, HALOS, and CALOS, and covering the neonatal population of entire gestational age groups.

## Methods

### Settings and infection control methods

Twenty-five tertiary hospitals participated in the current study. Among the hospitals, twenty-three tertiary hospitals were located in Shandong province which involving 13 major cities, one tertiary hospital in Hebei province and the other one in the Inner Mongolia Autonomous Region. Nineteen hospitals were general hospitals and 6 were maternal and child health care hospitals.

Because all 25 hospitals have their own maternity/obstetric ward, most neonates were born on site and only a few were transferred. The number of beds ranged from 20 to 60. The ratio of nurses to bed ranged from 0.4 to 1.2 and physician to nurse ranged from 0.3 to 0.5. The bed occupancy rate was maintained above 90%. All 25 hospitals have an infection control committee. Trained infection control nurses were available at all units at all times. All NICUs had a hand hygiene policy, but no audits of staff compliance were undertaken. Alcohol-based hand rub solutions and disinfectant dispensers filled with betadine 7.5% were provided at hand-wash sinks, and clean disposable tissue papers for hand-drying were sufficiently available. None of 25 NICUs had laminar flow devices. Surveillance cultures were only used when an outbreak was suspected but were not routinely undertaken.

### Identification and susceptibility testing

Blood cultures were performed for any infant presenting with clinical signs or symptoms of sepsis according to the local guidelines of each hospital. Blood samples were collected by trained nurses or physicians. Venipuncture sites were prepared with 75% isopropyl alcohol, followed by iodine tincture, and then wiped with alcohol. Skin site was allowed to dry for 1 min prior to venipuncture. A general policy of using one culture bottle exclusively for newborns with at least 1 ml of blood sample was adopted by all hospitals. The sample was delivered to the microbiology laboratory within 2 h of collection by staff members. Training of blood culture collection procedures were undertaken regularly in local hospitals. Each microbiology laboratory performed routine microbiology tests, including organism identification and antimicrobial susceptibility testing (AST). Blood cultures were performed at recruited hospital laboratories and incubated using Bactec FX system (Becton Dickinson, USA) in 15 hospitals and BacT/ALERT 3D system (bioMérieux, France) in 10 hospitals. Automated methods include use of VITEK-2 compact system in 23 hospitals and Vitek-MS system in 2 hospitals for organism identification and AST. Manual methods include organism identification by agar plate and biochemical workup and AST by disk diffusion methods. AST of pathogens was undertaken according to Clinical and Laboratory Standards Institute guidelines [15, 16].

### Definitions

Inclusion criteria of infants: infants gestational age ≥ 37 weeks with sepsis occurred within 28 days after birth and infants gestational age < 37 weeks with sepsis occurred within the corrected age of 44 weeks [14, 17, 18].

Our definition of neonatal sepsis was formulated with consideration to Chinese consensus of diagnosis and treatment. Neonatal sepsis was defined as the growth of at least a single pathogen (bacterium or fungus) from the blood of an infant who fulfilled all three of the following criteria: (1) One or more of the following infection-related clinical manifestations: respiratory distress, apnea; tachycardia or bradycardia; systemic hypotension or hypoperfusion; hypothermia or fever (T > 38.5℃ or < 36℃); convulsions, hypotonia, irritability or lethargy; feeding intolerance or intestinal obstruction. (2) One or more abnormal hematologic index: white blood cell count (< 5 × 10^9/L or > 30 × 10^9/L for age ≤ 3d or > 20 × 10^9/L for age > 3d), increase of immature/total neutrophil (≥ 0.16 for age < 3d or ≥ 0.12 × 10^9/L for age ≥ 3d), C-reactive protein level (≥ 10 mg/L) or abnormal procalcitonin level (≥ 0.5 mg/L). (3) Antibiotics used for at least 5 days [19–21].

Contaminants were defined based on the following criteria: (1) isolates usually considered as contaminants (eg, *Micrococcus* species); (2) *CoNS* in the absence of a peripheral or central catheter when the blood samples was collected; (3) a mixed flora of *CoNS* was cultured; (4) isolates considered as contaminants by the neonatologist, implying that antibiotics used less than 5 days [14].

EOS was defined as the occurrence of sepsis at or before the first 72 h of life while LOS was defined as the occurrence of sepsis after the first 72 h of life. Among LOS, infants with sepsis onset ≤ 48 h after admission were considered as having CALOS, and those with onset > 48 h after admission were considered as having HALOS [14].

Repeatedly isolated pathogens were regarded as identical BSI episodes unless they occurred beyond 7 days after the last positive culture result [22]. Antimicrobial susceptibilities were reported as susceptible or resistant (intermediate or resistant) based on microbiology reports. Resistance proportions were reported as number of resistant pathogens/number of pathogens tested.

Multidrug-resistant (MDR) gram-negative (GN) bacteria were defined isolates tested against at least 1 agent in 3 or more of the following antimicrobial categories: carbapenems (imipenem and meropenem), penicillins (piperacillin, Ampicillin, and piperacillin/tazobactam), broad-spectrum cephalosporins (ceftazidime and cefepime), monobactams (aztreonam), aminoglycosides, and fluoroquinolones [23].

All-cause mortality was defined as a proportion of neonates deceased among admitted neonates [24].

### Data collection and statistical analyses

This study is a retrospective, multicenter case series of hospitalized neonates with positive blood cultures. The medical record of each infant with positive blood culture was reviewed by a local neonatologist and the data was recorded onto a unified standardized worksheet from all 25 NICUs. Data of the worksheet included the medical institution, number of cots, staffing ratios, medical record number, gestational age, birth weight, gender, date of birth, date of blood cultures obtained, isolates identified, clinical significance of isolates and antimicrobial susceptibility. Other clinical data were also collected including body temperature, heart rate, white blood cell count, procalcitonin and C-reactive protein in the 72 h before and after blood cultures were collected. Worksheets from 25 NICUs were sorted out critically by a neonatologist and a clinical microbiologist was involved in the interpretation of these microbial results. The ethics committees of all 25 participating hospitals approved the study and allowed data sharing. Procedures were in accordance with the Helsinki Declaration of the World Medical Association.

Statistical analysis was performed using the *SPSS* software version 25.0 (*SPSS* Inc, Chicago, IL). Descriptive analysis was performed to characterize the study population and pathogens. Categorical data are presented as percentages, numerical data as median with 25th and 75th percentiles (interquartile range, IQR). The univariable logistic regression was used to evaluate group differences in all-cause mortality and fungal BSI. Two-sided *P* < 0.05 indicated significance.

## Results

**Fig. 1:**
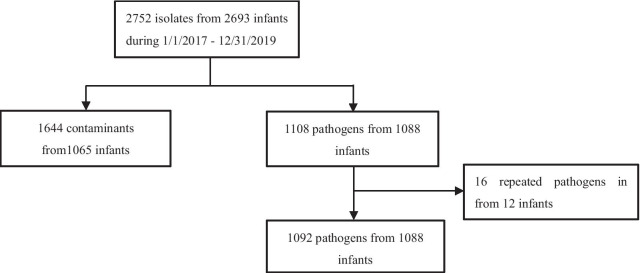
Study flow diagram

A total of 2752 isolates from 2693 infants were obtained from 25 NICUs between January 1, 2017 and December 31, 2019. However, only 39.7% (1092/2752) of isolates were classified as disease causing pathogens that met inclusion criteria, excluding 1644 (59.8%) contaminants and 16 (0.5%) repeated pathogens (Fig. 1). Of these 1092 pathogens, 349 (32%) pathogens were responsible for EOS, 702 (64.3%) for HALOS and 41 (3.7%) for CALOS. No neonate experienced both EOS and HALOS. Four infants had *Klebsiella pneumoniae* caused by polymicrobial pathogens, namely one with *Escherichia coli* and *Klebsiella pneumoniae*, one with *Klebsiella pneumoniae* and *Enterococcus* species, and two infants with *Pseudomonas aeruginosa* and *Klebsiella pneumoniae*. The characteristics of the study population are presented in Table 1. Of the infants studied, 57.4% (624/1088) were male. All-cause mortality was 7.4% (26/349) in EOS and 4.4% (31/698) in HALOS. No infants with CALOS died. All-cause mortality was higher among neonates with EOS than HALOS (7.4% vs. 4.4%, [OR] 0.577, 95% CI 0.337–0.989; *P* = 0.045). Table 2 shows the pathogen distribution that caused neonatal EOS, HALOS and CALOS. GN bacteria was the commonest in both EOS and HALOS, with the proportion of 56.7% (198/349) and 62.2% (437/702), respectively.

**Table 1:** Demographic and clinical characteristics of the study population

	All patients (n = 1088 infants)	EOS (n = 349 infants)	HALOS (n = 698 infants)	CALOS (n = 41 infants)
Birth weight (grams), n (%)				
< 1500	352 (32.4)	65 (18.6)	287 (41.1)	0
1501–2500	230 (21.1)	81 (23.2)	148 (21.2)	1 (2.4)
≥ 2500	506 (46.5)	203 (58.2)	263 (37.7)	40 (97.6)
Gestational age (weeks), n (%)				
< 28	104 (9.6)	23 (6.6)	81 (11.6)	0
28–34	386 (35.5)	87 (24.9)	299 (42.8)	0
34–37	130 (11.9)	61 (17.5)	65 (9.3)	4 (9.8)
≥ 37	468 (43)	178 (51)	253 (36.2)	37 (90.2)
Male sex, n (%)	624 (57.4)	184 (52.7)	441 (63.2)	29 (70.7)
Age during blood sampling (days) (median, IQR)	10 (2–22)	1 (0–2)	17 (10–27)	13 (9–23)
Length of hospital stay (days) (median, IQR)	36 (15–56)	17 (10–36)	39 (19–63)	16 (11–24)
All-cause mortality (%)	57 (5.3)	26 (7.4)	31 (4.4)	0

*IQR* interquartile range

**Table 2:** Pathogen Distributions in EOS, CALOS and HALOS at 25 NICUs, January 2017–December 2019

Pathogens	EOS (*n* = 349 in 349 infants) *n* (%)	HALOS* (*n* = 702 in 698 infants) *n* (%)	CALOS (n = 41 in 41infants) n (%)	Total (n = 1092 in 1088 infants) n (%)
Gram-positive bacteria	149 (42.7)	175 (25.0)	36 (87.8)	360 (33.0)
*CoNS*	28 (8.0)	81 (11.5)	0	109 (10.0)
*GBS*	51 (14.6)	15 (2.1)	19 (46.3)	85 (7.8)
*Staphylococcus aureus*	19 (5.4)	35 (5.0)	17 (41.5)	71 (6.6)
*Enterococcus spp.*	11 (3.2)	18 (2.6)	0	29 (2.7)
*Listeria monocytogenes*	22 (6.3)	1 (0.1)	0	23 (2.1)
Other Gram-positive bacteria	18 (5.2)	25(3.6)	0	43 (4.0)
Gram-negative bacteria	198 (56.7)	437 (62.2)	5 (12.2)	640 (58.6)
*Escherichia coli*	95 (27.2)	110(15.7)	4 (9.8)	209 (19.1)
*Klebsiella pneumoniae*	31 (8.9)	196 (27.9)	0	227 (20.8)
*Enterobacter spp.*	19 (5.4)	50 (7.1)	1 (2.4)	70 (6.5)
*Serratia marcescens*	19 (5.4)	19 (2.7)	0	38 (3.5)
*Acinetobacter baumannii*	9 (2.6)	28 (4.0)	0	37 (3.4)
*Pseudomonas aeruginosa*	5 (1.4)	16 (2.3)	0	21 (2.0)
Other Gram-negative bacteria	20 (5.8)	18 (2.6)	0	38 (3.5)
*Fungi*	2 (0.6)	90 (12.8)	0	92 (8.4)
*Candida albicans*	2 (0.6)	37 (5.3)	0	39 (3.6)
*Candida parapsilosis*	0	18 (2.6)	0	18 (1.6)
*Candida glabrata*	0	10 (1.4)	0	10 (0.9)
*Candida guilliemondii*	0	8 (1.1)	0	8 (0.7)
*Candida tropicalis*	0	3 (0.4)	0	3 (0.3)
*Other fungi*	0	14 (2.0)	0	14 (1.3)

*Four infants had HALOS caused by polymicrobial pathogens, namely one with *Escherichia coli* and *Klebsiella pneumoniae*, one with *Klebsiella pneumoniae* and *Enterococcus species,* and two infants with *Pseudomonas aeruginosa* and *Klebsiella pneumoniae*

### Early-onset sepsis

**Fig. 2:**
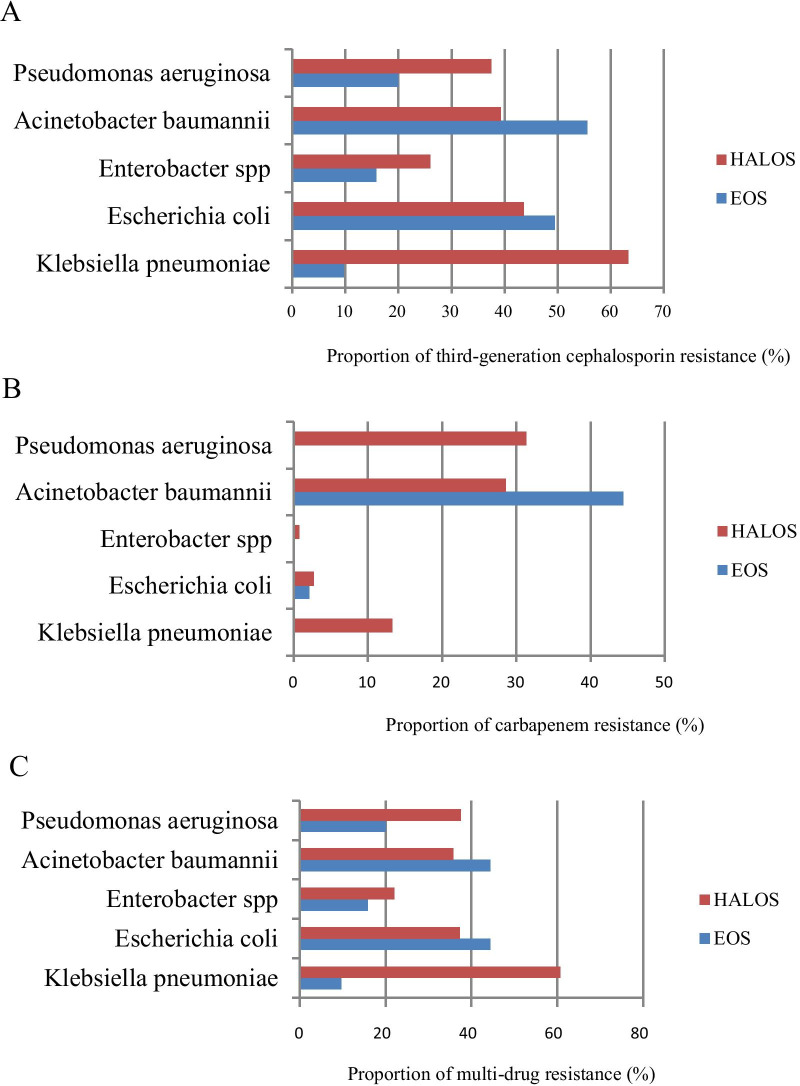
**A** Proportion of third-generation cephalosporin resistant main GN bacteria in EOS and HALOS. **B** Proportion of carbapenem resistant main GN bacteria in EOS and HALOS. **C** Proportion of multi-drug resistant main GN bacteria in EOS and HALOS;

In EOS, 51% (178/349) were term infants and 58.2% (203/349) were neonates with normal birth weight. Overall, *Escherichia coli* and *GBS* were the most common pathogenic bacteria of EOS, accounting for 27.2% (95/349) and 14.6% (51/349). 84.3% (43/51) of *GBS* were identified from term infants and 15.7% (8/51) in preterm infants with EOS. In contrast, *Escherichia coli* were responsible for 61.1% (58/95) of pathogens in preterm infants, and 38.9% (37/95) in term infants with EOS. In maternal and child health hospital, *Escherichia coli* (19.4%; 24/124) and *Klebsiella pneumoniae* (16.1%; 20/124) were the most common pathogenic bacteria of EOS, followed by *GBS* (10.5%; 13/124). In contrast, the top three pathogens in general hospital were *Escherichia coli* (31.6%; 71/225), *GBS* (16.9%; 38/225) and *Listeria monocytogenes* (7.1%; 16/225). Carbapenem resistance was uncommon in EOS: 44.4% (4/9) of *Acinetobacter baumannii* isolates and 2.1% (2/95) of *Escherichia coli* were resistant (Fig. 2). The proportion of resistant isolates was highest for *Escherichia coli* in EOS: 84.9% (79/93) were ampicillin resistant, 49.5% (47/95) were third-generation cephalosporins resistant and 44.4% (42/95) were multidrug resistant (Fig. 2).

### Hospital-acquired LOS

Pathogens responsible for HALOS were more common in neonates with gestational age 28–34 weeks (42.8%; 299/698), followed by > 37 weeks (36.2%; 253/698). Data from HALOS patients noted 41.4% (287/698) were very low birth weight (< 1500 g) neonates and 37.7% (263/698) neonates with normal birth weight. *Klebsiella pneumoniae*, the leading pathogen of HALOS, was responsible for 27.9% (196/702) of the cases, followed by *Escherichia coli* (15.7%, 110/702) and *Fungi* (12.8%, 90/702). *Klebsiella pneumoniae* was primarily identified among preterm infants (75.5%; 148/196). *Klebsiella pneumonia* was the top common pathogen both in general hospital (23%; 121/527) and in maternal and child health hospital (42.9%; 75/175). *Escherichia coli* (17.3%; 91/527) and *Fungi* (14*.*2%; 75/527) were the second and third common pathogen in general hospital respectively, whereas *CoNS* (14.3%; 25/175) and *Escherichia coli* (10.9%; 19/175) was in a second and third place in maternal and child health hospital. Most GN bacteria in HALOS showed a high degree of antimicrobial resistance, not only to commonly used ampicillins (87.5–100%) and third-generation cephalosporins (26–63.3%) but also to reserved antibiotics such as carbapenems (2.7–31.3%) (Fig. 2). A high proportion of *Klebsiella pneumoniae* (60.7%; 119/196), *Escherichia coli* (37.3%; 41/110), *Pseudomonas aeruginosa* (37.5%; 6/16) and *Acinetobacter baumannii* (35.7%; 10/28) in HALOS were multidrug resistant. An outbreak of four carbapenem-resistant *Klebsiella pneumoniae* strains was presented in a general hospital without further molecular typing. Among gram-positive (GP) bacteria in HALOS, methicillin resistance was detected in 77.8% (63/81) of *Coagulase-negative staphylococci* (*CoNS*) and 80% (28/35) of *S. aureus*. Significant methicillin resistance rate was identified in *S. aureus* which was 88.2% (15/17) (Additional file 1: supplement I). All the isolates of *CoNS*, *Staphylococcus aureus* and *GBS* were susceptible to vancomycin, but three *enterococci* isolates (16.7%, 3/18) in HALOS were resistant which were from three different NICUs.

For *Fungi*, 30.4% (28/92) were detected from maternal and child health hospital and 69.6% (64/92) from general hospital. 97.8% (90/92) of *Fungi* cases were identified in HALOS. *Candida albicans* (42.4%; 39/92) and *Candida parapsilosis* (19.6%; 18/92) were the top two species isolated (Table 2). All *Fungi* were sensitive to 5-fluorocytosine, amphotericin B and voriconazole. *Candida albicans* strains were resistant to both fluconazole (3.3%; 3/92) and itraconazole (3.3%; 3/92). *Fungal* BSI occurred in 28 of 405 infants in 8 NICUs (6.9%) with routine use of antifungal prophylaxis, whereas 64 in 683 infants in the other 17 NICUs (9.4%) with using antifungal drugs only in necessity. No significant difference were observed on *fungal* BSI based on whether or not routine use of antifungal prophylaxis in NICUs (9.4% vs 6.9%, [OR] 0.718, 95% CI 0.452–1.140; *P* = 0.161).

### Community-acquired LOS

In CALOS, 97.6% (40/41) of pathogens were detected from neonates with normal birth weight and 90.2% (37/41) from term infants (Table 1). In contrast, 87.8% (36/41) of pathogens that caused CALOS were GP bacteria. *GBS* and *Staphylococcus aureus* were responsible for 46.3% (19/41) and 41.5% (17/41) of CALOS cases. None of the five GN pathogens causing CALOS were carbapenem and multidrug resistant. High rates of resistance were observed in *GBS* in CALOS to erythromycin (89.5%, 17/19) and clindamycin (80%, 15/19) (Additional file 1: supplement I).

### Differences in neonatal period and beyond

The majority of pathogens were identified during 28 days of life (84.3%, 921/1092). The most common pathogens during 28 days of life were *Escherichia coli* (21.7%, 200/921), *Klebsiella pneumoniae* (19%, 175/921) and *CoNS* (9.1, 84/921). In preterm, *Klebsiella pneumoniae* (30.4%, 52/171), *Fungi* (18.1%, 31/171) and *CoNS* (14.6%, 25/171) were the top three pathogens after 28 days of life in hospitalization. Carbapenem resistance rates in *Klebsiella pneumoniae* were 12% (21/175) and 9.6% (5/52), and multidrug resistance rates 56.6% (99/175) and 42.3% (22/52) during 28 days of life and after 28 days of life in hospitalization, respectively. Five carbapenem resistant *Escherichia coli* and vancomycin resistant *Enterococcus spp.* were all identified during 28 days of life. No difference was found about all-cause mortality during 28 days of life and after 28 days of life in hospitalization (5.7% vs 2.9%, [OR] 0.505, 95% CI 0.199–1.282; *P* = 0.151).

## Discussion

Due to different empirical uses of antibiotics and implementation of preventive measures, the pathogens involved in neonatal BSI may vary geographically and temporally [9]. The results state that *Escherichia coli*, *Klebsiella pneumoniae* and *GBS* were the leading causes, respectively. This study was conducted in China, a lower-middle income country with paucity of high-quality data. The most common organism causing HALOS was *Klebsiella pneumoniae* and approximately 2/3 of those isolates were MDR or resistance of third-generation cephalosporin. The result would be important for clinicians in Chinese NICUs to guide optimal clinical prevention strategies and for consideration of empirical antibiotic treatment during clinical management.

The proportion of all positive blood cultures judged to be contaminants in our study was nearly 60% which was much higher than the proportion of 13–56% reported from western countries [25–27]. Reports from 12 British hospitals revealed approximately half blood culture judged to contaminants in 5 hospitals compared with no more than a quarter in the other 7 hospitals [28]. This is striking, nevertheless, few relevant data was reported in China. Standardizing blood culture collection methods, optimizing blood volume, creating checklists, and reinforcing nurse education were verified to develop a best practice for reduction of blood culture contamination [29].

Nosocomial infection is a major health problem particularly in NICUs in developing countries [30]. In current study, about two thirds of pathogens identified were responsible for HALOS. Approximately 60% of HALOS were due to GN bacteria which was similar to the results from previous Chinese studies [11, 12]. A review of 11,471 bloodstream samples indicated that GN bacteria was detected from no less than 60% of positive blood cultures in all the developing settings of the world [31]. Similar to that reported from South Asia [3] and Egypt [7], *Klebsiella pneumoniae* was the most common GN bacteria causing LOS. By contrast, *CoNS* was the most common pathogen in western countries for LOS, such as 40% in Switzerland [14]. The preponderance of *CoNS* might indicate the developed regions’ adoption of neonates with lower gestational age and lower birth weight and prolonged use of central catheters which are risk factors for *CoNS* infection [32]. The predominance of GN bacteria in our developing counties may largely be attributed to the lack of standard infection-control practices. Insufficient hand hygiene, lack of essential equipment and supplies including sinks, running water and disposables, overcrowding and understaffing are described to be key contributors to nosocomial infection caused by GN bacteria [33]. A recent prospective population-based cohort study reported HALOS frequently correlated with low gestational age, low birth weight and comorbidities. The study population in our cohort appears similar to that previously reported [14]. Therefore, implementation of these basic hygiene practices should be emphasized more in Chinese NICUs to minimize the hazards of the high incidence of HALOS caused by GN bacteria. Also, empiric antibiotics selected to treat suspected HALOS in Chinese NICUs need to effectively treat GN bacteria, especially *Klebsiella pneumoniae*.

Differences in all-cause mortality between EOS and HALOS may be partially explained by the prophylactically antibiotics prescribed and unrestricted use of broad-spectrum and advanced antibiotics in our NICUs, although it is highly discouraged. For part of neonates diagnosed with EOS, born with shock, disseminated intravascular coagulation and multiple organ failure, they had poor outcomes frequently even with active treatment.

In the current study, no neonate experienced both EOS and HALOS. This may be due to the fact that infants with EOS were less likely to be of low birth weight and lower gestational age, which may have resulted in them being discharged after a short course of antibiotics, avoiding an excessive length of stay and prolonged hospital exposure and interventions.

Another remarkable finding in the Chinese NICUs was the relatively high percentage of *Fungi* in HALOS. Nearly all the identified *Fungi* infections were responsible for HALOS, which was paralleled with other Chinese studies [11, 12]. Similarly, recent studies have also reported outbreaks of *fungal* nosocomial infection in Chinese NICUs [34, 35]. In China, huge variation existed among NICUs in the use of antifungal prophylaxis. In a recent multicenter study, antifungal drugs were prescribed in 20% of LOS and 35% of fungal LOS, including prophylaxis or empirical treatment [12]. Prolonged antibiotic therapy, broad spectrum antibiotic exposure may be the connection to high prevalence of *fungal* nosocomial infections [36]. This highlights the need to develop new and more effective approaches to prevent HALOS.

A strength of this study was that all 25 hospitals have their own maternity/obstetric ward and almost all neonates were born onsite with only a few transferred. This makes the pathogens in EOS more precise and representative. In the current study, *Escherichia coli* was the most common pathogen in EOS, followed by *GBS*. Similarly, the latest surveillance from a national neonatal research network in US demonstrated the shift from *GBS* to *E. coli* as the leading pathogen and the increase in *Escherichia coli* infections among very low-birth-weight infants [37]. *Escherichia coli* was the most common pathogen among preterm infants in current study which was similar to those reported in most developed countries [6, 14, 37]. Previously, *GBS* was reported to be a rare cause of EOS and was documented in only a few reports from China and other Asian countries [7, 38]. The reason for the low proportion of *GBS* in EOS may be partially due to the overuse of antenatal antibiotics in China. However, we did not have the data on antenatal antibiotic use. Invasive *GBS* infections in Chinese neonates are supposed to be rare because of the lower rate of *GBS* colonization in pregnant women and the higher protective antibody concentrations in mothers, so that screening and preventive measurements have been suspended [38]. *Escherichia coli* was the most common pathogen in EOS and the second common in HALOS. The extraordinary similarity of this spectrum supports the assumption that the cause of EOS may not only be due to vertical transmission from mothers but also can be caused by unsanitary practices in the labour rooms and NICUs. Further investigations are of urgent need to identify the causes of GN bacteria in EOS in China and subsequently develop targeted prevention strategies.

Most CALOS occurred in term or near-term newborns, accounting for a small percentage of BSI. *GBS* and *Staphylococcus aureus* were the leading responsible pathogens. Similar to reports in developed counties, infants with CALOS had a higher birth weight and gestational age and fewer adverse outcomes compared to those who developed HALOS [14]. The relatively low incidence rate may be related to the lack of strict management and relatively easy access to antibiotics in China. Antibiotics may have been administered before admission resulting in false negative results in blood cultures.

Our study showed that isolation frequency and AMR of GN bacteria differed significantly between EOS and HALOS. Bacteria strains isolated in HALOS were more resistant to ampicillin, gentamicin, third-generation cephalosporin and carbapenem evaluated in our study compared to those isolated in EOS. Studies from both developing and developed countries have also shown similar findings [3, 20]. This may suggest that bacteria strains associated with vertical transmission are different to those that are nosocomial transmission and supports the idea that different antimicrobial regimens are needed.

In our study, a concerning feature of the HALOS pathogens is the high resistance rates in *Klebsiella pneumoniae* to the third-generation cephalosporins and carbapenems, and the proportion of multi-drug resistant strains. Notably a multi centre South Asia study involving nosocomial infections reported resistance rates in *Klebsiella pneumoniae* of 71.3–73.7% to cefotaxime and 9.4–11.5% to meropenem. Multidrug resistance was reported in 66.1–75.3% of *Klebsiella pneumoniae* [3]. In a Taiwanese study, the overall proportion of GN infection was comparable to that in our study, and K. pneumoniae was the most common isolate, but the rate of carbapenem resistance (18.6%) was higher than our rate [39]. Also, they found that the most frequent mechanism of MDR GN bacteria was ESBL production [39]. Ampicillin/piperacillin and third-generation cephalosporin are the first choices for empirical treatment to neonatal sepsis in China [40]. Unrestricted use of broad-spectrum cephalosporin may explain the high drug resistance rates in *Klebsiella pneumoniae* and makes the choice of antibiotics extremely difficult.

In general, the severe situation of high antimicrobial resistance rate in HALOS is probably multifactorial and may include lack of standardized infection control policies, higher rates of broad-spectrum antibiotics use and the low nurse-to-bed ratios observed in NICUs.

Strengths of this study include its enrollment of pathogens from all neonates admitted to the NICU, distinguishing pathogens and AMR between EOS, HALOS and CALOS, and large sample size. The following limitations should be considered: (1) the choice of a definition on neonatal sepsis is a limitation inherent to many studies. The diagnosis of neonatal sepsis is mainly based on Chinese Consensus formulated by Chinese Pediatric Society which may not have been widely accepted [30]. The diagnosis of infection of *CONS* usually relies on confirmation with second blood culture, but this practice is not routinely followed in LMICs. Therefore we are not certain whether *CONS* represented true pathogen infections or potential contaminants; (2) antibiotics are usually administered to neonates born offsite with suspected sepsis, which could impair recovery of pathogens associated with CALOS. Neonates who were discharged and present with CALOS at a later stage are not necessarily admitted to the tertiary facility where they were born, and as such, may have been omitted from this study; (3) we merely collected the data on the proportion of causative pathogens between EOS, HALOS and CALOS, but we were unable to calculate the incidence rates.

## Conclusion

In this study, EOS and HALOS were most commonly caused by GN bacteria, with *Escherichia coli* and *Klebsiella pneumoniae* being the major pathogens, respectively. Only a small proportion of pathogens were identified for CALOS, most commonly *GBS*. A high proportion of the pathogens isolated were due to HALOS, and the prevalence of AMR was high. Effective interventions are urgently needed to reduce HALOS in LMICs. Continued surveillance is warranted to identify pathogen distribution and AMR, and to distinguish between EOS, HALOS and CALOS causing agents.

## Supplementary Information

**Additional file 1.** Antimicrobial Resistance of Common EOS and HALOS Causing Pathogens at 25 NICUs, January 2017 - December 2019.

## Data Availability

The data that support the findings of this study are available from the corresponding authors upon reasonable request.

## Abbreviations

BSI: Neonatal bloodstream infection
AMR: Antimicrobial resistance
GBS: Group B streptococcus
EOS: Early-onset sepsis
HALOS: Hospital-acquired late-onset sepsis
CALOS: Community-acquired late-onset sepsis
GN: Gram negative
GP: Gram positive
LMICs: Low-income and middle-income countries
CONS: Coagulase negative staphylococcus
MDR: Multidrug-resistant
AST: Antimicrobial susceptibility testing
