# Supplementary material for: Pathogens distribution and antimicrobial resistance in bloodstream infections in twenty-five neonatal intensive care units in China, 2017–2019
Source: Antimicrob Resist Infect Control. 2021 Aug 16;10:121. doi: 10.1186/s13756-021-00989-6 (PMC8365905; doi:10.1186/s13756-021-00989-6)
Supplement: Supplementary file 1 — Additional file 1. Antimicrobial Resistance of Common EOS and HALOS Causing Pathogens at 25 NICUs, January 2017 - December 2019. [file 13756_2021_989_MOESM1_ESM.docx]

**Supplement 1** Antimicrobial Resistance of Common EOS and HALOS Causing Pathogens at 25 NICUs, January 2017–December 2019

|  | EOS  (No. isolates resistant/ no. tested (%)) | HALOS  (No. isolates resistant/ no. tested (%)) |
| --- | --- | --- |
| *Klebsiella pneumoniae*  Ampicillin  Third-generation cephalosporin  Gentamicin Piperacillin/Tazobactam Carbapenem  Multidrug resistance | 24/30 (80)  3/31 (9.7)  2/31 (6.5)  3/30 (10)  0  3/31 (9.7) | 174/186 (93.5)  124/196 (63.3)  41/196 (20.9)  38/194 (19.6)  26/196 (13.3)  119/196 (60.7) |
| *Escherichia coli*  Ampicillin  Third-generation cephalosporin  Gentamicin Piperacillin/Tazobactam Carbapenem  Multidrug resistance | 79/93 (84.9)  47/95 (49.5)  31/95 (32.6)  2/95 (2.1)  2/95 (2.1)  42/95 (44.4) | 95/106 (89.6)  48/110 (43.6)  20/110 (18.1)  5/107 (4.7)  3/110 (2.7)  41/110 (37.3) |
| *Enterobacter spp*  Ampicillin  Third-generation cephalosporin  Gentamicin Piperacillin/Tazobactam Carbapenem  Multidrug resistance | 14/19 (73.7)  3/19 (15.8)  1/19 (5.3)  1/19 (5.3)  0  3/19 (15.8) | 42/48 (87.5)  13/50 (26)  3/50 (6)  4/50 (8)  4/50 (8)  11/50 (22) |
| *Acinetobacter baumannii*  Ampicillin  Third-generation cephalosporin  Gentamicin  Piperacillin/Tazobactam Carbapenem  Multidrug resistance | 6/9 (66.7)  5/9 (55.6)  3/9 (33.3)  4/9 (44.4)  4/9 (44.4)  4/9(44.4) | 25/27 (92.6)  11/28 (39.3)  4 /28 (14.3)  6 /28 (28.6)  8/28 (28.6)  10/28 (35.7) |
| *Pseudomonas aeruginosa*  Ampicillin  Third-generation cephalosporin  Gentamicin  Piperacillin/Tazobactam Carbapenem  Multidrug resistance | 3/4 (75)  1/5 (20)  0  0  0  1/5 (20) | 14/14 (100)  6/16 (37.5)  1/16 (6)  5/16 (31.3)  5/16 (31.3)  6/16 (37.5) |
| *CoNS*  Methicillin  Vancomycin | 18/28 (64.3)  0 | 63/81 (77.8)  0 |
| *Staphylococcus aureus*  Methicillin  Vancomycin | 1/19 (5)  0 | 28/35 (80)  0 |
| *GBS*  Clindamycin  Erythromycin  Vancomycin | 38/50 (76)  42/51(82.4)  0 | 11/15(73.3)  12/15(80)  0 |
| *Enterococcus spp.*  Vancomycin | 0 | 3/18 (16.7) |
